# Current Guidelines for the Implementation of Flywheel Resistance Training Technology in Sports: A Consensus Statement

**DOI:** 10.1007/s40279-023-01979-x

**Published:** 2024-01-04

**Authors:** Marco Beato, Kevin L. de Keijzer, Alejandro Muñoz-Lopez, Javier Raya-González, Marco Pozzo, Björn A. Alkner, Antonio Dello Iacono, Jordi Vicens-Bordas, Giuseppe Coratella, Sergio Maroto-Izquierdo, Oliver Gonzalo-Skok, Stuart A. McErlain-Naylor, Fernando Martin-Rivera, Jose L. Hernandez-Davo, Luis Suarez Arrones, Rafael Sabido, Moises de Hoyo, Rodrigo Fernandez-Gonzalo, Lena Norrbrand

**Affiliations:** 1 School of Allied Health Sciences, University of Suffolk, Ipswich, UK; 2 Departamento de Motricidad Humana y Rendimiento Deportivo, University of Seville, Seville, Spain; 3 Faculty of Sport Sciences, University of Extremadura, Cáceres, Spain; 4 Master de Fútbol, Universidad Pablo de Olavide, Seville, Spain; 5 SmartCoach Technologies, Inc., Seville, Spain; 6 Department of Orthopaedic Surgery, Eksjö, Region Jönköping County, Sweden; 7 Department of Biomedical and Clinical Sciences, Linköping University, Linköping, Sweden; 8 School of Health and Life Sciences, University of the West of Scotland, Paisley, Scotland; 9 Sport, Exercise, and Human Movement (SEaHM), University of Vic-Central University of Catalonia, Barcelona, Spain; 10 Sport and Physical Activity Studies Centre (CEEAF), University of Vic-Central University of Catalonia, Barcelona, Spain; 11 Department of Biomedical Sciences for Health, Università degli Studi di Milano, Milan, Italy; 12 i+HeALTH, European University Miguel de Cervantes (UEMC), Valladolid, Spain; 13 Department of Communication and Education, Universidad Loyola Andalucía, Seville, Spain; 14 School of Sport, Exercise and Health Sciences, Loughborough University, Loughborough, UK; 15 Research Group in Prevention and Health in Exercise and Sport, University of Valencia, Valencia, Spain; 16 Faculty of Health Sciences, Isabel I University, Burgos, Spain; 17 Department of Sport Sciences, Universidad Pablo de Olavide, Seville, Spain; 18 Sport Research Centre, Department of Sport Sciences, Miguel Hernández University, Elche, Spain; 19 Department of Physical Education and Sport, University of Seville, Seville, Spain; 20 Performance Department, Aston Villa Football Club, Birmingham, UK; 21 Department of Laboratory Medicine, Division of Clinical Physiology, Karolinska Institutet, Stockholm, Sweden; 22 Unit of Clinical Physiology, Karolinska University Hospital, Stockholm, Sweden; 23 Division of Environmental Physiology, School of Engineering Sciences in Chemistry, Biotechnology and Health, KTH Royal Institute of Technology, Solna, Sweden

## Abstract

**Background:**

Flywheel resistance training has become more integrated within resistance training programs in a variety of sports due to the neuromuscular, strength, and task-specific enhancements reported with this training.

**Objective:**

This paper aimed to present the consensus reached by internationally recognized experts during a meeting on current definitions and guidelines for the implementation of flywheel resistance training technology in sports.

**Methods:**

Nineteen experts from different countries took part in the consensus process; 16 of them were present at the consensus meeting (18 May 2023) while three submitted their recommendations by e-mail. Prior to the meeting, evidence summaries were developed relating to areas of priority. This paper discusses the available evidence and consensus process from which recommendations were made regarding the appropriate use of flywheel resistance training technology in sports. The process to gain consensus had five steps: (1) performing a systematic review of systematic reviews, (2) updating the most recent umbrella review published on this topic, (3) first round discussion among a sample of the research group included in this consensus statement, (4) selection of research group members—process of the consensus meeting and formulation of the recommendations, and (5) the consensus process. The systematic analysis of the literature was performed to select the most up-to-date review papers available on the topic, which resulted in nine articles; their methodological quality was assessed according to AMSTAR 2 (Assessing the Methodological Quality of Systematic Review 2) and GRADE (Grading Recommendations Assessment Development and Evaluation). Statements and recommendations scoring 7–9 were considered *appropriate*.

**Results:**

The recommendations were based on the evidence summary and researchers’ expertise; the consensus statement included three statements and seven recommendations for the use of flywheel resistance training technology. These statements and recommendations were anonymously voted on and qualitatively analyzed. The three statements reported a score ranging from 8.1 to 8.8, and therefore, all statements included in this consensus were considered *appropriate*. The recommendations (1–7) had a score ranging from 7.7 to 8.6, and therefore, all recommendations were considered *appropriate*.

**Conclusions:**

Because of the consensus achieved among the experts in this project, it is suggested that practitioners and researchers should adopt the guidelines reported in this consensus statement regarding the use of flywheel resistance technology in sports.

## Key Points

**Table:** 

Although the eccentric phase is frequently the focus of flywheel training, not all exercises, users, or training loads achieve eccentric overload. Consequently, practitioners should define this resistance method as ‘flywheel resistance exercise or training’ instead of ‘eccentric overload’.
Reliable flywheel training exercise outputs are contingent upon the user’s effort, training experience (i.e., familiarization), moment of inertia (kg^.^m^2^) selected, and the mechanical characteristics of the devices used.
Practitioners can use flywheel resistance training as a valid method to develop chronic morphological adaptations in both sporting and healthy male or female populations.
Practitioners can use flywheel resistance training as a valid method to increase mechanical power and jump performance of male and female populations. Enhancements can be seen with interventions that are short and consisting of lower weekly training frequencies.
Practitioners can use flywheel resistance training as a valid method to increase athletes’ ability to perform sport-specific acceleration and deceleration actions.

## Introduction

The concept of using the inertia of spinning flywheels to generate resistance was used by Hill more than 100 years ago [1], and about 30 years ago the gravity-independent flywheel exercise device was developed for use in space to counteract muscle loss in astronauts during long-duration space flights [2]. In fact, resistance training using flywheel devices has been shown to counteract quadriceps muscle atrophy during 90 days of bedrest [3], and even induce hypertrophy during unilateral lower limb suspension [4]. The unique loading principle, allowing for variable loading within a repetition, also made the device interesting for terrestrial use. Thus, the first report of positive injury prevention outcomes with flywheel training was published 20 years ago [5]. Since then, numerous studies have been published involving athletes from various sports, demonstrating that flywheel resistance training can generate significant morphological and neuromuscular adaptations [6–10].

Flywheel devices allow for maximal or near maximal voluntary forces throughout each repetition of a set [2, 11]. To initiate movement, the participant must pull, push, or curl a cord/strap connected to a fixed shaft that holds the flywheel disc(s). The force applied unwinds a strap connected to the shaft of the device, which starts to rotate. Once the whole range of motion of the concentric phase is performed, the cord/strap rewinds and the person must resist the pull of the rotating flywheel disc(s) by performing an eccentric muscle action [4, 11–13]. If performed appropriately, this allows for a greater application of force during the eccentric action and thereby a mechanical eccentric overload (i.e., greater eccentric than concentric peak force or power) as well as greater muscle activation [6, 13–15]. Apart from technique, it is important that the device and analysis of mechanical outputs are appropriate to optimize flywheel training [16]. The combination of repetitive maximal concentric phases and increased demands during the eccentric phase of movements may enhance physiological and mechanical adaptations and benefit athletes. Flywheel training may elicit a preferential upregulation of satellite cell activity and transcriptional pathways in fast-twitch muscle fibers, increase protein synthesis and ultimately stimulate muscular hypertrophy [17]. These effects seem to justify the acute and chronic enhancements seen with flywheel training programs.

Flywheel resistance training has become more integrated within resistance training programs in a variety of sports due to the neuromuscular, strength, and task-specific enhancements reported with this training [18–20]. Flywheel resistance training has been effectively implemented within post-activation performance enhancement protocols to acutely enhance sport performance [17, 21–23] and for chronically enhancing strength and sport performance [8, 20, 24]. A recent review conducted by Raya-González et al. [25] concluded that flywheel training may generate quicker adaptations (e.g., strength and power) than traditional resistance training programs. Moreover, flywheel training has also improved jumping [26, 27], linear sprint [28, 29], and change of direction [19, 30] performance, which are all key variables for success in sport [18]. More recently, some recommendations for periodization of flywheel training in team sports have been provided to support the integration of flywheel training without interfering with regular sport training [31] as well as to monitor and decrease muscular asymmetries in sport [32]. The aforementioned findings highlight that flywheel training provides a valid and safe strength training method for athletic and healthy populations to enhance sport performance and strength.

Although the body of literature available on flywheel resistance exercise has increased in recent years, we still have some relevant issues when such research is interpreted [17]. For instance, we have several review papers that have investigated the effect of flywheel resistance training on sport parameters, but these reviews frequently use the same studies (e.g., the same randomized controlled trials) to reach their conclusions [13, 19, 24], which may lead to repetitive and consequent amplification of errors derived from some of the original papers (e.g., methodological or statistical errors that lead to incorrect data interpretation) [33]. Moreover, the use of some specific terms within flywheel resistance training research is not accurate or consistent, and this is an issue that needs to be addressed. Furthermore, it has been found that on many topics there are some differences between practitioners’ perceptions and research evidence about the effect of flywheel resistance training in sport [34]. Hence, an overall consensus among researchers has not yet been reached. Given the importance of using appropriate flywheel resistance training, an internationally recognized consensus-based standard is necessary to set some specific recommendations for the use of flywheel resistance technology. The aim of this paper is to present the consensus reached by internationally recognized researchers (experts) during a meeting on current definitions and guidelines for the implementation of flywheel resistance technology in sports.

## Methods

### Consensus Process

The first step of this consensus statement was to synthesize and analyze the current state of the literature. Following a systematic search of PubMed/Medline and SPORTDiscus (only articles published in English before 30 January 2023 were included), a total of 22 reviews or systematic reviews were found. Authors (MB and KDK) reviewed all the reviews published thus far and identified the most comprehensive and up to date review, which was *“The effect of flywheel training on strength and physical capacities in sporting and healthy populations: an umbrella review”* [20]. This umbrella review followed procedures to reduce the impact of limitations of individual reviews and meta-analyses. Moreover, it allowed for the synthesis and appraisal of the existing evidence and thereby comparison of conclusions based on all relevant published data. Finally, this type of review allows for an analysis of possible or existing bias in the literature that can negatively affect the validity and applicability of the scientific evidence [35]. This umbrella review was selected because it is one of the latest and the most comprehensive reviews on flywheel resistance training. In addition to this, the umbrella review summarized 11 reviews previously published and scored their methodological quality according to the Assessing the Methodological Quality of Systematic Review 2 (AMSTAR 2) and Grading Recommendations Assessment Development and Evaluation (GRADE) [36, 37] criteria. However, some of these selected articles were narrative reviews and were not suitable for this consensus statement procedure.

*Step 1: Systematic review of the systematic reviews*

As reported, this consensus statement was initially based on a previous umbrella review [20], which was updated by removing any narrative reviews as well as adding any new existing systematic reviews. To do so, a new systematic search was performed using the following approach.

The systematic review of the systematic reviews (SROSR) was performed according to systematic review guidelines and followed the Preferred Reporting Items for Systematic Reviews and Meta-analyses (PRISMA, 2020) statement guidelines [38].

### Systematic Search

PubMed search: (flywheel exercise) OR (flywheel inertia) OR (flywheel resistance training) OR (flywheel resistance exercise) OR (variable inertial) OR (rotary inertial) OR (inertial training) OR (inertial exercise) OR (isoinertial training) OR (isoinertial exercise) OR (eccentric overload) OR (eccentric overload training) OR (enhanced eccentric) OR (gravity independent) OR (flywheel training) AND (sport performance [MeSH]) OR (muscular strength).

Filters applied: Full text, Meta-Analysis, Systematic Review, English.

SportDiscus search: (flywheel exercise) or (flywheel inertia) or (flywheel resistance training) or (flywheel resistance exercise) or (variable inertial) or (rotary inertial) or (inertial training) or (inertial exercise) or (isoinertial training) or (isoinertial exercise) or (eccentric overload) or (eccentric overload training) or (enhanced eccentric) or (gravity independent) or (flywheel training) or (AND (sport performance [MeSH]) OR (muscular strength).

Four new reviews were found; one was a scoping review [39], two were narrative reviews [25, 40], and one was a systematic review with meta-analysis [41].

*Step 2: Update of the umbrella review by De Keijzer et al., 2022 *[20]

**Fig. 1:**
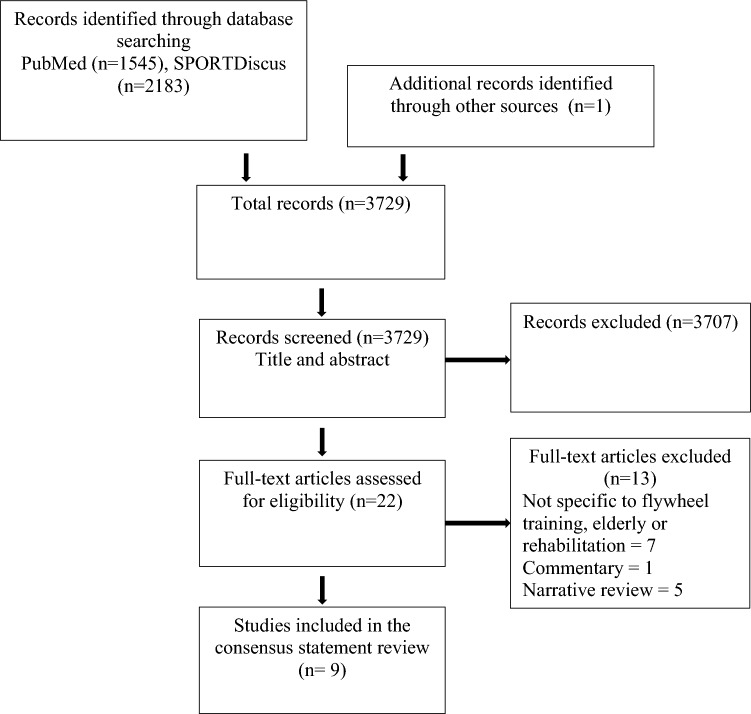
Flow diagram of the study retrieval process

The inclusion and exclusion criteria previously used [20] were followed for this SROSR with the addition that only systematic reviews and meta-analyses could be considered for this project. Therefore, three narrative reviews included by De Keijzer et al. [20] were excluded in this SROSR [9, 15, 18]. A more recent literature search was performed (Fig. 1).

As stated above, three new reviews, specifically a scoping review [39] and two narrative reviews [25, 40], were excluded because of the inclusion criteria used in this SROSR. However, the authors (MB and KDK) reviewed the excluded papers to examine whether these articles could have influenced the validity of this SROSR. Based on our evaluation, these reviews would not substantially change the final evidence reported in this SROSR as well as the final recommendations and summary assessment of this consensus statement. One new systematic review with meta-analysis was included in this SROSR [41].

### Quality Assessment

The eight systematic reviews previously published and subsequently analyzed by De Keijzer et al. [20] were integrated with the most recent one [41], while the narrative reviews analyzed in the previous umbrella review were removed (only systematic reviews and meta-analyses were included in the current study). A new analysis of the methodological quality according to the AMSTAR 2 and GRADE was therefore performed. Once again, the final output following this procedure takes the name of SROSR and includes nine reviews [8, 13, 19, 24, 41–45].

### Results of the Systematic Review of Systematic Reviews

AMSTAR 2 is a tool that allows for the assessment of methodological quality of systematic reviews [36]. In this case, we reported that seven reviews were considered *moderate* and two were rated as *high* quality, while none of the reviews were considered *low* quality [36]. GRADE is a transparent framework for developing and presenting summaries of evidence and provides a systematic approach for making clinical practice recommendations [37]. Using these GRADE principles, one review was rated of *moderate* quality (authors believe the true effect is probably close to the estimated effect), six of the nine reviews were considered of *high* quality (authors have a lot of confidence that the true effect is like the estimated effect), while two reviews did not critically appraise the included primary studies and were therefore not assigned a GRADE rating.

*Step 3: First round discussion among a sample of the research group included in this consensus statement*

The results of the SROSR were circulated amongst a sample of the members of the research group defined as the ‘leading group’, which consisted of the following researchers: MB, KDK, LN, AML and JRG. The invitation to be a member of the leading group was sent by email on 9 January 2023. The first meeting amongst the members of the leading group was on 6 February 2023. These researchers assessed the first three stages of the process before sharing their findings of the SROSR and starting a critical analysis with the whole research group (who were selected and invited to stage 4).

*Step 4: Selection of research group members—process of the consensus meeting and formulation of the recommendation*

### Selection of Research Group Members

Prior to initiating this research, the leading group set the criteria to identify potential expert group members. Researchers included in this project were selected based on their publication record (i.e., to be or to have been researchers with a minimum of five published peer-reviewed articles in the field of flywheel resistance technology) and to have practically applied flywheel training (i.e., use of flywheel resistance technology in applied sport settings). Potential group members were contacted via e-mail asking them if they were interested in taking part in the consensus statement. The researchers included in the first stage of the recruitment invited new potential researchers to be included in this research group. The leading group completed Step 4 on 8 March 2023 when the new potential researchers were invited to collaborate on this consensus statement. An email asking for their availability to collaborate on this project was sent on 10 March and a reminder on 17 March 2023.

### Researcher/Expert Group Demographics

All consensus meeting participants were researchers and experts in the field of flywheel resistance technology in sports. Areas of expertise among the participants included strength and conditioning, sports science, and sports medicine. The years of experience, geographic locations, and gender of the experts were also recorded.

### Areas of Priority

Following this expression of interest and the final selection of the members, the following key areas were identified as priorities for consensus: definition of flywheel resistance technology, characteristics of flywheel resistance technology, exercise load monitoring, flywheel training periodization, flywheel training for hypertrophy, strength and power development, flywheel training for sprinting and change of direction performance, and flywheel training for injury prevention.

*Step 5: Consensus process*

The included expert group members were contacted via email asking for their availability to meet the rest of the leading group for an online meeting to discuss the consensus statement on 18 May 2023. The results of the SROSR and the first draft of the recommendations were emailed to each expert group member at least 1 week before the meeting. During the meeting, members reported their observations, and the final recommendations were made following an open discussion. The researchers that did not take part in the meeting e-mailed their feedback and suggestions to the leading group, who integrated this feedback into the final recommendations. Experts that did not participate in the meeting and did not submit their feedback were excluded from the final authors’ list. Following the conclusion of the meeting and when the recommendations were made, the expert group members voted on the final recommendations of this consensus statement. This process was conducted in an anonymous format, following the procedure and scoring system used in a previous consensus statement [46]. The score was performed using a 9-point Likert scale, where 1 was the minimum and 9 was the maximum. Overall, scores from 1–3 were considered and defined as *inappropriate*, scores of 4–6 were considered *uncertain*, and scores of 7–9 were considered *appropriate*. The final consensus statement was written based on the evidence reported in the SROSR and expert group members’ votes.

## Results

Twenty-five potential experts were contacted by email and 19 of them completed the survey and took part in this project. Researchers included in this research were selected based on their previous publications on the topic and their experience as flywheel practitioners. Researchers were identified (multiple selections were allowed) as experts in strength and conditioning (*n* = 13), sports science (*n* = 17), medical doctors (*n* = 1), and/or biomechanics (*n* = 1). Experts had a mean of 12 (range: 4–26) years of experience in the use of flywheel resistance technology. Their geographical location was Europe (*n* = 19). The participants identified as men (*n* = 18) and a woman (*n* = 1).

The consensus meeting took place on 18 May 2023 in an online format. Of the 19 experts involved in this process, 16 took part in this event while three submitted their recommendations by e-mail to the leading group. Therefore, all experts were included in the consensus voting process, which was concluded on 23 June 2023, and they were listed as authors of this paper.

**Fig. 2:**
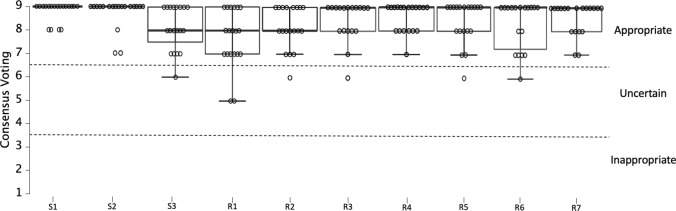
The consensus voting statement and qualitative analysis of the recommendations. The score is reported with a 9-point Likert scale, where 1 is the minimum and 9 is the maximum. Overall, scores of 1–3 are considered and defined as *inappropriate*, scores of 4–6 are considered *uncertain*, and scores of 7–9 are considered *appropriate*. *R* recommendation, *S* statement

The consensus voting statement and qualitative analysis of the recommendations are reported in Fig. 2.

## Discussion

The aim of this consensus statement was to present a consensus reached by internationally recognized researchers (experts) during a meeting on current definitions and guidelines for the implementation of flywheel resistance training technology in sports. The consensus-based standard set some specific statements and recommendations for the use of flywheel resistance technology.

*Statement 1: Flywheel resistance training is characterized by the use of rotating flywheel discs or cones to provide resistance. The concentric action is initiated by pulling the strap connected to the shaft of the device, spinning (accelerating) the flywheel/cone. Once the strap rewinds around the shaft, an eccentric action is performed to decelerate the flywheel/cone rotation.*

The inertia of a flywheel or cone provides the resistance when using flywheel resistance technology [20]. The concentric action is initiated by pulling a strap connected to the shaft, setting the flywheel/cone into rotation [13]. When the strap rewinds around the shaft, an eccentric action (braking force) decelerates the flywheel/cone rotation [47, 48]. Hence, the training method is characterized by increasing or decreasing the stored kinetic energy of a rotating flywheel or cone [6]. The intensity of the movement can be adjusted by changing the flywheels or the cone, and thereby changing the moment of inertia. It usually consists of one or more stacked discs or a cone with radially mounted masses.

During the concentric phase, the kinetic energy can be calculated as follows:

$$ E_{k} = \raise.5ex\hbox{$\scriptstyle 1$}\kern-.1em/ \kern-.15em\lower.25ex\hbox{$\scriptstyle 2$} I\omega^{2} $$

where *E*_*k*_ = kinetic energy (J), *I* = flywheel moment of inertia (kg⋅m^2^), and *ω* = angular velocity (rad/s).

After accelerating the flywheel in the concentric phase, the user applies a braking eccentric force to the strap, bringing the flywheel to a complete stop. Without considering friction, which dissipates work into heat, work done (i.e., change in kinetic energy) during the concentric and eccentric phases is equal.

*Statement 2: Eccentric overload is a term frequently misunderstood and misused by researchers and practitioners. Although the eccentric phase is frequently the focus of flywheel training, not all exercises, users, or training loads achieve eccentric overload. Consequently, practitioners should define this resistance method as ‘flywheel resistance exercise or training’ instead of ‘eccentric overload’.*

Eccentric overload is defined as a higher mechanical output during the eccentric phase compared with the concentric phase and is considered a relevant characteristic of flywheel resistance exercise training [17, 22, 49, 50]. Previously, it was a common misconception for researchers and practitioners to generalize that flywheel resistance exercise consistently obtains eccentric overload [49]. This was because flywheel resistance training was quite a new training method and there was not much research available. This technology has undergone significant evolution in recent years, with various updates (e.g., rotary encoders), which have provided practitioners with the ability to measure more variables and better control training than what was previously possible.

We acknowledge that differences in exercises, moments of inertia, training experience, and braking techniques likely influence whether eccentric overload is achieved [14, 49–52]. Moving forward, practitioners should define this resistance method as ‘flywheel resistance exercise or training’. Additionally, if researchers or practitioners would like to discuss eccentric overload, they need to confirm it numerically (eccentric: concentric ratio > 1). Specifically, they should report the parameters monitored (i.e., power, speed, force) and whether they used average or peak values. Ideally, the reliability of measures should also be analyzed and reported when discussing mechanical outputs and their respective ratios.

In flywheel resistance exercise, power is one of the most common parameters analyzed and it can be calculated as follows:

$$ P = E_{k} /t $$

where *P* = power (W), *E*_*k*_ = kinetic energy (J), and *t* = time (s).

Power is also expressed as.

$$ P = T \cdot \omega $$

where *P* = power (W), *T* = torque (Nm), and *ω* = angular velocity (rad/s).

*Statement 3: Reliable flywheel training exercise outputs are contingent upon the user’s effort, training experience (i.e., familiarization), moment of inertia (kg* ^**.**^ *m* ^*2*^ *) selected, and the mechanical characteristics of the devices used. To obtain the best training response, the user should focus on the production of (near) maximal effort during each repetition and on the timing/technique of the braking force in the eccentric action, which allow for (near) maximal muscle activation and intensity of the workout.*

From a training perspective, although different flywheel devices are present in the current market [14, 52, 53], flywheel resistance technology allows for a high level of torque over the entire concentric phase and parts of the eccentric phase. Moreover, flywheel resistance devices allow for maximal, or near maximal, muscle activation during the workout [11, 12, 54, 55]. Because of the unique characteristics of flywheel exercise, the amount of torque, power produced, and muscle activation depend on the exercise execution, exercise selection, and the user’s experience [17, 55–57]. Furthermore, the manipulation of technique (e.g., assisted vs unassisted), braking strategies (e.g., at the end of the eccentric phase), biomechanics in the specific exercise (e.g., optimal joint angles for force production), and the mechanical variables monitored (e.g., peak power values) play a crucial role in the eccentric output and also in the determination of the presence of an eccentric overload (see Statement 2) [50, 51].

The torque required to achieve any given angular acceleration or deceleration of the flywheel system (disc/cone + shaft) increases proportionally to the flywheel system’s overall moment of inertia:

$$ T = I \cdot \alpha, $$

where *T* = torque (Nm), *I* = moment of inertia (kg⋅m^2^), and α = angular acceleration (*rad/s*^*2*^). This moment of inertia can be increased via increases in the mass and/or radius of the attached discs or the flywheel device itself (moment of inertia = mass × radius^2^, summed for every point mass). The linear force required by the user to generate this torque about the flywheel device’s axis is inversely proportional to the wrapping radius of the strap around the shaft:

$$ F = T / r, $$

where F = force (N), *T* = torque (Nm), and *r* = radius (*m*). Additionally, the smaller the wrapping radius, the more revolutions of the flywheel that will be required for any given linear displacement of the user/attachment. For these reasons, the exercise resistance therefore depends not only on the moment of inertia of attached discs, but also on the characteristics of the devices used (i.e., mass, radius, and shape) [58–60]. Flywheel devices using the same disc moment of inertia will produce different resistances if the shaft radii are different.

In conical shaft devices, the instantaneous radius changes significantly along the range of motion. It is worth noting that, in practice, this effect is also observed in cylindrical shaft machines, for example, due to the strap recoiling on itself. In flywheel devices, therefore, the maximum diameter (at the beginning of concentric action, with the strap fully recoiled) is variable; it depends on the strap length (which in turn depends on the range of motion, subject height, etc.) and on the recoil tension force during the previous repetition. In short, radius variation along the range of motion is present in any flywheel device. This makes it difficult and unreliable to compute the force from torque (which is attained from measured angular speed and inertia). Therefore, it is more accurate to use power as the main measured variable, and refrain from introducing force unless this can be reliably measured directly.

*Recommendation 1: Practitioners can use linear and rotary encoders to monitor mechanical outputs (i.e., power, velocity, and force) and design inertia-power, inertia-velocity, and inertia-force curves.*

In weightlifting or with weight stack machines, the maximum repetition obtained with a certain weight is commonly used as the benchmark for the training design (i.e., intensity monitoring). However, there is no load that represents this concept in flywheel resistance technology [14]. Practitioners can monitor and calculate several mechanical variables in flywheel resistance training (e.g., using linear and rotary encoders) such as peak and mean power, velocity, and force that allows them to design inertia-power, inertia-velocity and inertia-force curves [49, 61] that could be used to individualize training programs. Practitioners can manipulate the moment of inertia to increase or decrease the mechanical outputs (e.g., speed and power) to achieve their training goals [31, 49, 61]. However, practitioners should be aware that the existence of a familiarization procedure (before testing) and previous flywheel resistance training experience play a key role in the reliability of mechanical outputs during training. Therefore, an adaptation period with the machines before proceeding with the design of profiles (e.g., inertia power) or tests (peak power) is strongly recommended [49, 61–63].

*Recommendation 2: Further research should include specific detail around any periodization model (if present) and training plan used in intervention studies to offer insights about the benefits of their use. The current literature is not strong enough to define evidence-based recommendations.*

Based on the updated literature review and the opinions of experts involved in this consensus statement, it is difficult to draft conclusions and definitive recommendations around the use of specific periodization models involving flywheel resistance technology. It is clear that periodization plays a key role in long-term physical development [64, 65]. However, the evidence currently available around flywheel resistance training periodization is quite limited and is mostly based on experts’ opinions [31]. To date, no research suggests any specific periodization model (e.g., linear, undulating, or block periodization) is superior to others. Therefore, several factors such as sport type, athlete’s level, and experience should be considered when determining which periodization model is most appropriate.

Based on the current evidence concerning flywheel training (SROSR), it is possible to recommend some simple guidelines that practitioners could follow; adequate familiarization assuring appropriate technique and experience is needed to optimize concentric and eccentric outputs and eccentric overload [8, 66, 67]. The application of flywheel training 2–3 times per week can enhance muscular force and mass in shorter periods (4–6 weeks); however, longer periods (> 10 weeks) are likely to be necessary to induce greater adaptations [43, 47]. During an initial training period of 10 weeks, practitioners could adopt a linear-periodization model and progressively increase intensity and/or volume [20, 47]. However, more sophisticated strategies should be adopted after this initial period (> 10 weeks). In some sports (e.g., football), the time available for resistance training is quite limited. Therefore, practitioners may be limited to restricted training frequency, specifically one or two sessions a week, with a volume ranging from 1 to 6 sets of 5–10 repetitions as was reported in 11 studies analyzed in a recent systematic review [19]. Because several factors should be considered when designing a periodization model, practitioners should evaluate the current evidence available (SROSR) and should use it in conjunction with their practical experience to develop the most appropriate training programs for athletes.

*Recommendation 3: Practitioners can use flywheel resistance training as a valid method to develop chronic morphological adaptations in both sporting and healthy male or female populations. Flywheel training can generate some hypertrophic adaptations in short training periods (from 4 to 8 weeks), with a training frequency usually between two and three sessions a week.*

The importance of hypertrophy for both sport performance and health reasons is well documented [68, 69]. Flywheel resistance training has been proposed as a valid method to develop hypertrophy in both sporting and sedentary populations [6, 9, 70]. In contrast with traditional weight training, flywheel resistance technology allows for approximately maximal muscle activation throughout the concentric action and during a part of the eccentric action, throughout all repetitions of a set [4, 12, 55]. The evidence reported in the reviews included in our SROSR suggest flywheel resistance training can be used to develop chronic morphological adaptations such as hypertrophy in both male [13] and female [8] populations. Regarding male populations, it seems that flywheel training can generate great hypertrophic adaptations in short training periods (from 4 to 8 weeks), where results of ≥ 5% increases of muscle volume, cross-sectional area, and mass have been reported [6, 71–73]. Commonly, flywheel resistance training studies have used a training frequency of between two and three sessions a week [25], although one study reported some adaptation with a lower training frequency of one session per week [30]. The majority of studies reported in the literature focused on lower limbs, while not much knowledge is currently available regarding the effect of flywheel resistance training for developing hypertrophy of the upper limbs [25]. Regarding intensity, the majority of studies use moments of inertia > 0.05 kg^.^m^2^ [25]. Regarding female populations, practitioners can select a large range of moments of inertia (0.025–0.14 kg^.^m^2^) to achieve desired muscular adaptations [8]. Based on the current literature, it seems that higher moments of inertia should be preferred to lower moments of inertia to stimulate muscular hypertrophy [17]; in particular, high loads and slow exercises would favor the increment of time under tension (time under activation during a set and high loading in the eccentric action), which should favor hypertrophic adaptations. However, clear guidelines regarding intensity, volume, and training frequency cannot be defined because these training parameters should be selected in accordance with factors including the age, sporting level, and previous training experience (e.g., elite female players or sedentary elderly). In most cases, we suggest practitioners progressively increase the moment of inertia and volume of exercises (multi-set exercises can generate greater adaptations than single-set exercises) to obtain a progressive overload (see Recommendation 2). A further point that is worth remembering is that the existence of eccentric overload during the exercise does not influence the subsequent increase in muscle mass, as reported in a systematic review [24]. Therefore, flywheel resistance training could be prescribed with or without eccentric overload if the main aim is to develop muscular hypertrophy. It is likely that the absolute demands of the exercise are more important than the relative comparison of concentric and eccentric phases.

*Recommendation 4: Practitioners can use flywheel resistance training as a valid method to develop chronic strength adaptations in both sporting and healthy male or female populations. Moreover, flywheel resistance training elicits improvements in strength development with different testing methodologies (i.e., isokinetic, isotonic) and muscular contractions (i.e., concentric and eccentric).*

An athlete’s strength is determined by components such as their morphological (e.g., cross-sectional area) and neuromuscular characteristics. It is well known that strength training is a critical factor for improving sport performance and reducing injury risk [74, 75]. However, strength adaptations can be assessed in several ways. For instance, it is possible to take into account improvements in a specific part of the force–velocity curve [18], during a specific phase (e.g., concentric or eccentric) of a muscular contraction [44], or assessing a specific type (e.g., isotonic or isokinetic) of contraction [52]. Based on the coaches’ aims, it is possible to tailor the characteristics of the training program to target specific strength improvements that are suitable for the sport population of interest. Based on the current literature on flywheel resistance training, we have seen that only a few weeks (4–6 weeks) are necessary to generate strength improvements (assessed in various ways), with a training frequency of 2–3 sessions per week and a volume in the range of 2–4 sets of 7–10 repetitions for the lower limbs and with similar volumes for the upper limbs [18]. However, practitioners should consider that the strength improvements following flywheel training are very closely related to the previous strength level of the population trained. For instance, very large improvements (maximal voluntary contraction of 11%–12% during concentric and eccentric phases) were found after only 5 weeks of training in sedentary subjects [4], while smaller improvements were found in football populations [19]. Regarding improvements in maximal strength (i.e., 1-repetition maximum), a recent review found that flywheel training protocols (half squat exercise) lasted usually between 6 and 8 weeks, consisting of 4 sets of 7 repetitions, with a moment of inertia from 0.050 to 0.11 kg·m^2^ [18]. Similarly, flywheel leg extension training improved lower limb maximal voluntary isometric contraction following a protocol with durations of 4–5 weeks, with a weekly frequency of 2–3 sessions per week, and a moment of inertia of 0.090 kg·m^2^ [18]. However, the use of specific volumes and intensities (moment of inertia) should not be too generalized because several factors related to the participants (e.g., sport populations, age of the athletes, previous familiarization with the technology) and the machines (see Statement 3) can impact the outcomes of the training program and so should be considered by coaches when flywheel training programs are designed [16, 59]. Coaches should therefore use the moment of inertia based on the mechanical characteristics of their devices and the sport population that they want to train. Finally, it was reported that the existence of eccentric overload during exercises can offer advantages for chronic enhancement of muscular force, something that practitioners should consider [76].

*Recommendation 5: Practitioners can use flywheel resistance training as a valid method to increase mechanical power and jump performance of male and female populations. Enhancements can be seen with interventions that are short and consisting of lower weekly training frequencies. However, further research is needed to clearly define the dose response using flywheel resistance training—especially when considering differences in response between populations. Finally, flywheel resistance exercise can be effectively implemented within post-activation performance enhancement protocols to acutely enhance sport performance.*

Implementation of resistance training methods such as flywheel training is likely to enhance jumping performance and mechanical power [13, 19, 43, 45]. Improvements in power and jump performance are likely to be associated with enhanced stretch–shortening cycle function and optimized ability to repeatedly perform high-intensity eccentric actions [77, 78]. The most up to date evidence (considered *moderate* and *high* quality) amongst male populations highlights that flywheel training interventions of 5–24 weeks enhance jumping performance [45]. Specifically for male soccer athletes, evidence of a *moderate* and *high* quality suggests flywheel training protocols (1–2 sessions per week; lasting 6–10 weeks) involving squats, lateral squats, or lunges can significantly enhance jump performance [19]. While one intervention (2 sessions per week; lasting 6 weeks) with healthy females elicited *large* improvements in jump performance [72], other studies (1–2 sessions per week; lasting 6–24 weeks) did not enhance jump performance with a mixed-cohort of athletes [79]. Although evidence supporting the use of flywheel training for female athletes is limited, greater training frequency is likely to enhance mechanical power and jump performance [8]. It is important to highlight that the present synthesis of evidence on flywheel training amongst male and female athletes also involves non-elite populations (i.e., healthy adults) [8, 43]. The present evidence may therefore inappropriately represent how flywheel training may elicit changes in jump and power performance with elite athletes [8, 43, 45]. Based on the present evidence, squats performed on cylindrical and conical shaft flywheel devices using a variety of moments of inertia are likely to elicit favorable adaptations in power and jump performance [19]. Nonetheless, further research into exercise selection and training intensity are critical for optimizing training interventions to enhance power and jump performance.

Flywheel resistance exercise has been effectively implemented within post-activation performance enhancement protocols to acutely enhance sport performance [17, 21–23]. Previous research reported that flywheel squat, deadlift, cross-cutting step, and lunge acutely increased vertical jump, changes of direction, and isokinetic (i.e., hamstrings eccentric torque) performance in different populations [48, 52, 80, 81]. Moreover, flywheel cross-cutting step, leg extension, and squat acutely modified muscles’ contractile properties assessed by tensiomyography [48]. Practitioners can use different moments of inertia (e.g., 0.029–0.11 kg·m^2^), based on the exercise selected, and multi-set exercises (e.g., 2–3 sets) to enhance sport-specific performance [15, 17]. Regarding the post-activation performance enhancement time-window, acute fatigue is dominant in the early part of the recovery period (e.g., 30 s), while potentiation is dominant in the second part (e.g., after 3 min) [15]; thus it is suggested practitioners plan a recovery period between the flywheel post-activation performance enhancement protocol and the subsequent exercises to facilitate transfer effects on athletic performance [17].

*Recommendation 6: Practitioners can use flywheel resistance training as a valid method to increase athletes’ ability to perform sport-specific braking and accelerating actions. Indeed, the systematic use of flywheel training within training will enhance acceleration, deceleration, sprint, and change of direction ability in sporting populations. Further studies are needed to evaluate the dose–response relationship between flywheel training and sprint performance amongst sport populations that typically adopt a low resistance training frequency per week (e.g., football).*

Flywheel resistance training has been commonly used to improve sprint and change-of-direction ability in sport [18, 20]. A meta-analysis reported that both sprinting and change-of-direction ability increase following short training protocols (5–10 weeks) [45]. Flywheel training involves repetitive maximal concentric and eccentric contractions and may lead to sport-specific improvements by enhancing muscular size, muscular strength, and capacity to exert force during change-of-direction actions [17, 51]. Another meta-analysis (involving 11 studies) found that flywheel training improves change-of-direction performance (e.g., 180° change of direction) more so than control groups in professional team sports [42]. This was further confirmed by a recent systematic review with meta-analysis that reported that change of direction ability can be improved in < 12 weeks of training [41]. Another meta-analysis [19] that assessed male soccer players found that flywheel resistance training presents contrasting evidence regarding its efficacy for enhancing sprint performance. This could be related to the low training dose that is usually prescribed in soccer studies; for instance, most of the interventions used a training frequency of 1–2 sessions a week or with an overall low flywheel resistance training volume [19]. However, it is not possible to provide a definitive explanation of this due to the number of contextual factors that play a role in speed (and physical) training in football. In contrast, the same review confirmed findings of previous reviews stating that flywheel resistance training is suitable for enhancing change-of-direction ability in football players [19]. Therefore, the experts involved in this consensus statement, after the evaluation of the SROSR, suggest practitioners use flywheel resistance training to improve the braking and accelerating actions that athletes experience when performing changes of direction as well as to improve sprinting capacity in sporting populations. However, it is our opinion that a greater understanding of low-dose flywheel training for the enhancement of sprint performance in sport (e.g., football) populations is still necessary.

*Recommendation 7: Further well-designed intervention studies (i.e., randomized controlled trials) are needed to verify the ability of flywheel resistance technology-based training interventions to reduce the likelihood of muscular (e.g., hamstring) and articular (e.g., anterior cruciate ligament) injuries in sport populations; moreover, further research is needed to evaluate its validity as a rehabilitation tool following an injury.*

One of the main advantages of flywheel resistance training is the mechanical work that can be performed during the eccentric phase [9]. Eccentric training can stimulate specific neuromuscular and morphological adaptations [82], which can lead to a reduction in likelihood of lower limb injuries (e.g., hamstring) [83]. However, adaptations and related benefits of flywheel training span from the combination of both concentric and eccentric contractions [6, 25]. As previously reported, the concentric phase of the movement is an indispensable requirement of generating a demanding eccentric phase [17, 51, 55, 56]. Therefore, it is clear that the acute mechanical load generated during flywheel resistance exercises and the related chronic benefits are related to the combination of both actions [14, 28, 51].

Regarding injury prevention using flywheel resistance training devices, it was found that the use of one or two flywheel resistance exercises, using moments of inertia ranging from 0.05 to 0.145 kg·m^2^, volume of 6–8 repetitions for 3–6 sets, training 2 times per week, reduced lower limb injuries in soccer players [5, 7, 57]. Although some evidence exists, the current body of literature about the capacity of flywheel resistance training programs to actually reduce the likelihood of muscular injuries is quite limited. Moreover, little is also known regarding its validity within rehabilitation; a recent paper showed that flywheel resistance training can be successfully used in populations with patellar tendinopathy [84] but more research in this field is needed. On this basis, no flywheel-specific evidence-based guidelines can be provided to practitioners until this area is further explored. Nonetheless, we suggest practitioners integrate flywheel resistance training in a progressive and systematic manner (in the same way that other resistance training methods are used) to improve athletes’ strength and obtain training adaptations. Intensity and volume should therefore be progressed (ideally with monitoring of mechanical outputs) for both injury prevention and rehabilitation objectives [66]. We suggest combining different flywheel exercises (e.g., squats, leg curls) with other suitable training methods to enhance injury prevention programs rather than relying solely on one training methodology [32, 75, 85].

### Quality of Evidence (AMSTAR/GRADE)

Of the nine included reviews, two were systematic reviews and seven were systematic reviews with meta-analyses. While two reviews were considered of *high* quality, seven were considered of *moderate* quality using the AMSTAR 2 checklist (Table 1). Future studies should aim to provide explicit statements wherein methods are established a priori (item 2), justify study inclusion/exclusion (with rationale) (item 7), assess individual studies’ risk of bias (item 9), clearly report funding/conflicts of interest (items 10 and 16), and consider likelihood of publication bias (item 15) [36]. In accordance with the adapted GRADE principles, six reviews were considered *high* quality (> 2 high quality studies), one was considered *moderate* quality (> 1 high or > 2 moderate quality studies), while two did not critically appraise the included primary studies and were therefore not assigned a GRADE rating. Future reviews should aim to include *high* quality studies to enhance the conclusions of their research [20].

**Table 1:** Overall results of the AMSTAR 2 and GRADE recommendations for systematic reviews and meta-analyses

Study	1	2	3	4	5	6	7	8	9	10	11	12	13	14	15	16	AMSTAR 2	GRADE
Allen et al. (2021) [19]	Yes	No	Yes	Yes	Yes	Yes	Yes	Yes	Yes	No	n/a	n/a	Yes	Yes	n/a	Yes	Moderate	High
Nuñez et al. (2017) [24]	Yes	No	Yes	Yes	No	Yes	No	Yes	Yes	No	Yes	No	No	No	Yes	No	Moderate	n/a
Liu et al. (2020) [42]	Yes	No	Yes	Yes	Yes	No	Yes	Yes	Yes	No	Yes	Yes	Yes	Yes	Yes	Yes	High	High
Maroto-Izquierdo et al. (2017) [13]	Yes	No	Yes	Yes	Yes	Yes	Yes	Yes	No	No	Yes	No	No	Yes	No	Yes	Moderate	High
Petré et al. (2018) [43]	Yes	No	Yes	Yes	No	Yes	Yes	Yes	No	No	Yes	Yes	No	No	Yes	Yes	Moderate	Moderate
Raya-González et al. (2020) [18]	Yes	Yes	Yes	Yes	Yes	Yes	Yes	Yes	No	No	Yes	No	No	Yes	Yes	No	Moderate	High
Raya-González et al. (2021) [8]	Yes	No	Yes	Yes	Yes	Yes	Yes	Yes	No	No	n/a	n/a	Yes	Yes	n/a	No	Moderate	High
Vicens Bordas et al. (2018) [70]	Yes	Yes	Yes	Yes	Yes	Yes	Yes	Yes	Yes	No	Yes	Yes	Yes	Yes	Yes	Yes	High	n/a
Chaabene et al. (2022) [41]	Yes	No	Yes	Yes	Yes	Yes	Yes	Yes	Yes	No	Yes	Yes	No	No	No	Yes	Moderate	High

*AMSTAR 2* Assessing the Methodological Quality of Systematic Reviews 2, *GRADE* Grading of Recommendations, Assessment, Development, and Evaluations, *n/a* not applied

### Final Considerations and Practical Applications

This consensus statement presents for the first time a consensus reached by internationally recognized researchers (experts) during a meeting on current definitions and guidelines for the implementation of flywheel resistance technology in sports. Firstly, a systematic analysis of the literature was performed to select the most up to date review papers available on the topic, which resulted in nine articles [8, 13, 19, 24, 41–45]; this process can be found in the flow chart (Fig. 1). Secondly, the researchers involved in this project assessed the methodological quality of the reviews according to AMSTAR 2 and GRADE, which can be found in Table 1. Regarding these reviews, all of the review papers were considered of *moderate* or *high* quality (AMSTAR 2), and seven were considered of *moderate* or *high* quality when considering GRADE; following that, the reviews selected in this consensus statement were used to discuss the main areas of priority and subsequently to formulate the recommendations for flywheel resistance training. Based on the current scientific evidence and researchers’ expertise, the consensus statement included three statements and seven recommendations for the use of flywheel resistance training technology. These statements and recommendations were anonymously voted on and qualitatively analyzed (see Fig. 2). Statements 1, 2, and 3 reported an average score of 8.8 ± 0.3, 8.7 ± 0.4, and 8.1 ± 0.8, respectively; therefore, all statements included in this consensus were considered *appropriate*. The recommendations (1–7) reported scores of 7.7 ± 1.0, 8.2 ± 0.7, 8.4 ± 0.7, 8.6 ± 0.5, 8.4 ± 0.7, 8.3 ± 0.9, and 8.6 ± 0.6, respectively; therefore, all recommendations included in this consensus were considered *appropriate* (scores of 7–9). Because of the consensus achieved among the researchers (experts) involved in this project, it is suggested that practitioners and researchers should adopt the guidelines reported in this consensus statement regarding the use of flywheel resistance training technology in sports.

#### Limitations and Future Directions

This consensus statement is not without limitations. Firstly, the expert group was from Europe, limiting the cultural diversity of the group. Secondly, only one woman was present in this expert group. Future meetings should try to improve diversity and involve researchers, experts, athletes, and other stakeholders. The selection of the areas of interest regarding flywheel resistance training was made by the experts involved in this research, which means some form of bias (e.g., confirmation bias, reporting bias) could be present; future research should try to limit the effect of these biases. Another limitation is related to the researchers’ inclusion process, which was based on two criteria: the researcher’s publication record (i.e., a minimum of 5 published peer-reviewed articles in the field of flywheel resistance technology) and the researcher’s expertise in applied flywheel training (i.e., use of flywheel resistance technology in applied sport settings). The first inclusion criterion does not consider the quality and impact of these publications, which is a limitation. Lastly, some recommendations were made using the current evidence and the current experts’ opinions; therefore, these recommendations should be methodically updated when new high-quality pieces of evidence are produced (i.e., new systematic reviews and meta-analyses, high-quality randomized controlled trials).

## Conclusions

This consensus statement is the first paper of its type for flywheel resistance training technology in sports. The definitions and guidelines for the implementation of flywheel resistance training technology in sports were reached in this consensus statement from internationally recognized experts. Three statements and seven recommendations were voted on and qualitatively considered as *appropriate*. Practitioners and researchers who use flywheel resistance technology in sports settings should adopt the guidelines reported in this consensus statement. Nevertheless, more high-quality studies and systematic reviews are needed to further evaluate the validity of this technology in the field of resistance training in sports.

## Data Availability

This manuscript does not have associated data.
